# Cell membrane fluidity and ROS resistance define DMSO tolerance of cryopreserved synovial MSCs and HUVECs

**DOI:** 10.1186/s13287-022-02850-y

**Published:** 2022-05-03

**Authors:** Mitsuru Mizuno, Takahisa Matsuzaki, Nobutake Ozeki, Hisako Katano, Hideyuki Koga, Takanori Takebe, Hiroshi Y. Yoshikawa, Ichiro Sekiya

**Affiliations:** 1 Center for Stem Cell and Regenerative Medicine, Tokyo Medical and Dental University (TMDU), 1-5-45, Bunkyo-ku, Yushima, Tokyo 113-8510 Japan; 2 Division of Strategic Research and Development, Graduate School of Science and Engineering, Saitama University, 255, Shimo-okubo, Sakura-ku, Saitama City, Saitama 338-8570 Japan; 3 Institute of Research, Tokyo Medical and Dental University (TMDU), Tokyo, Japan; 4 Department of Applied Physics, Graduate School of Engineering, Osaka University, 2-1, Yamadaoka, Suita City, Osaka 565-0871 Japan; 5 Department of Joint Surgery and Sports Medicine, Graduate School, Tokyo Medical and Dental University (TMDU), 1-5-45, Bunkyo-ku, Yushima, Tokyo 113-8519 Japan; 6 Organoid Medicine Project, T-CiRA Joint Program, Fujisawa, Kanagawa Japan; 7 Division of Gastroenterology, Hepatology and Nutrition and Division of Developmental Biology, Cincinnati Children’s Hospital Medical Center (CCHMC), Cincinnati, OH USA; 8 The Center for Stem Cell and Organoid Medicine (CuSTOM), Cincinnati Children’s Hospital Medical Center, Cincinnati, OH USA; 9 Department of Pediatrics, University of Cincinnati College of Medicine, Cincinnati, OH USA; 10 Department of Chemistry, Saitama University, 255, Shimo-okubo, Sakura-ku, Saitama City, Saitama 338-8570 Japan

**Keywords:** Cell membrane fluidity, ROS resistance, Cryopreserve, Mesenchymal stem cells, Human umbilical vein endothelial cells

## Abstract

**Objectives:**

Synovial mesenchymal stem cells (MSCs) have high freeze–thaw tolerance, whereas human umbilical vein endothelial cells (HUVECs) have low freezing tolerance. The differences in cell type-specific freeze–thaw tolerance and the mechanisms involved are unclear. This study thus aimed to identify the biological and physical factors involved in the differences in freeze–thaw tolerance between MSCs and HUVECs.

**Materials and methods:**

For biological analysis, MSC and HUVEC viability after freeze-thawing and alteration of gene expression in response to dimethyl sulfoxide (DMSO, a cryoprotectant) were quantitatively evaluated. For physical analysis, the cell membrane fluidity of MSCs and HUVECs before and after DMSO addition was assessed using a histogram for generalized polarization frequency.

**Results:**

HUVECs showed lower live cell rates and higher gene expression alteration related to extracellular vesicles in response to DMSO than MSCs. Fluidity measurements revealed that the HUVEC membrane was highly fluidic and sensitive to DMSO compared to that of MSCs. Addition of CAY10566, an inhibitor of stearoyl-coA desaturase (*SCD1*) that produces highly fluidic desaturated fatty acids, decreased the fluidity of HUVECs and increased their tolerance to DMSO. The combination of CAY10566 and antioxidant glutathione (GSH) treatment improved HUVEC viability from 57 to 69%. Membrane fluidity alteration may thus contribute to pore-induced DMSO influx into the cytoplasm and reactive oxygen species production, leading to greater cytotoxicity in HUVECs, which have low antioxidant capacity.

**Conclusions:**

Differences in freeze–thaw tolerance originate from differences in the cell membranes with respect to fluidity and antioxidant capacity. These findings provide a basis for analyzing cell biology and membrane-physics to establish appropriate long-term preservation methods aimed at promoting transplantation therapies.

**Supplementary Information:**

The online version contains supplementary material available at 10.1186/s13287-022-02850-y.

## Introduction

Mesenchymal stem cells (MSCs) are a promising source for cell therapy; however, the development of a long-term preservation technology for processed cells is necessary for their widespread use. Cryopreservation is the most common method for long-term cell preservation [1, 2]. Synovial MSCs have high freeze–thaw tolerance and can maintain their chondrogenic differentiation potential even after freezing and thawing [3]. These cells, immediately after freezing and thawing, have the same treatment effect as cultured cells when administered to a knee osteoarthritis model [4]. However, it is not clear why MSCs retain this high freeze–thaw tolerance. On the contrary, various cell types with low freeze–thaw tolerance are known; these include human umbilical vein endothelial cells (HUVECs) [5]. HUVECs belong to the endothelial lineage and are expected to be used a cell source for liver [6] and cartilage regeneration [7]. However, to achieve long-term practical use and for developing long-term preservation technologies for cells with low freeze–thaw tolerance, the molecular mechanism underlying cell type-specific freeze–thaw tolerance needs to be clarified.

Cryoprotectants have been used to prevent the cell damage caused by cryopreservation. Among the commonly used cryoprotectants, dimethyl sulfoxide (DMSO) is the most common [8, 9]. DMSO addition is intended to increase the osmotic pressure in the cryopreservation solution, thereby causing loss of water from cells with reduced formation of ice clumps inside cells and damage to intracellular organelles [10–12]. In contrast, the toxicity of DMSO itself to cells has also been reported in several studies [13, 14]. DMSO toxicity is correlated with alterations in gene expression profiles and generation of reactive oxygen species (ROS) [15–17]. DMSO shows a wide range of toxicity depending on cell type, and this difference in toxicity may originate from the biological and physical characteristics of the cells, resulting in differences in freeze–thaw tolerance.

The cell membrane is the first contact interface for DMSO. Under physiological conditions, the cell membrane is in a highly fluid state with abundant lipids, which broadens the diversity of cellular functions. Fluidity is one of the representative physical properties of the cell membrane, and its values varies among cell types [18]. Membrane fluidity has been considered to contribute to fundamental cellular functions such as cell adhesion [19] and migration [20, 21]. Although membrane fluidity is an important factor that influences the cryopreservation quality of sperm [22, 23], the impact of membrane fluidity on the cryopreservation quality of various cells other than germ cells is not well assessed. Furthermore, the effect of DMSO on the membrane fluidity of somatic cells, especially synovial MSCs and HUVECs, has not been clearly evaluated.

Analyzing the effects of DMSO on two types of cells with different freeze–thaw tolerances may facilitate identification of biological and physical factors involved in the different freeze–thaw tolerance characteristics. Thus, this study aimed to identify the biological and physical factors contributing to the different freeze–thaw tolerance of MSCs and HUVECs.

## Materials and methods

### Synovial MSCs and HUVECs

This study was approved by the Medical Research Ethics Committee of Tokyo Medical and Dental University (M2017-142), and all subjects provided informed consent. The human synovium was harvested from the knees of six female donors (55–81 years) with osteoarthritis, during total knee arthroplasty. The synovial cells were cultured as previously reported [24]. Briefly, the synovium was digested in a solution of 3 mg/mL collagenase (Sigma-Aldrich Co. LLC, Merck KGaA, Darmstadt, Germany) at 37 °C. After 3 h, the digested cells were filtered through a 70 µm cell strainer (Greiner Bio-One GmbH, Frickenhausen, Germany). The cells were cultured in α-minimum essential medium (α-MEM; Thermo Fisher Scientific, MA, USA) supplemented with 1% antibiotic–antimycotic (Thermo Fisher Scientific) and 10% fetal bovine serum in a cell culture incubator (Astec Co., Ltd., Fukuoka, Japan) at 37 °C under 5% CO_2_.

HUVECs from four donors were purchased from Lifeline® Cell Technology (CA, USA) and maintained in Endothelial Cell Growth Medium 2 (PromoCell GmbH, Heidelberg, Germany).

### Freeze-thawing

Synovial MSCs and HUVECs (3 × 10^5^ cells) were suspended in 250 µL of 95% FBS and 5% DMSO (Fujifilm Wako Pure Chemical Corporation, Tokyo, Japan). Freezing and thawing were performed as follows: first, the tubes containing the cells were placed in a bio-freezing vessel (Bicell, Japan Freezer, Tokyo, Japan); second, the frozen cells were thawed using a frozen cell thawing device (ThawSTAR, BioLife Solutions, WA, USA); third, the cell suspensions were diluted to 1 mL by slow dropping of 750 µL cold medium and used for analysis without washing. The cells were stored at − 150 °C for at least 1 month before analysis.

### Quantification of live cells

Flow cytometry was performed using the CellEvent® Caspase-3/7 Green Flow Cytometry Assay Kit (Thermo Fisher Scientific) and SYTOX™ blue dead cell stain (Thermo Fisher Scientific) on a FACS Verse system (Becton, Dickinson and Company; BD, NJ, USA). Briefly, the cells were suspended at 3 × 10^5^ cells/mL and stained for 30 min at 37 °C with caspase-3/7 Green in the dark. FITC^−^V500^−^ cells were evaluated as live cells. These data were analyzed using FlowJo software (Tree Star Inc., CA, USA).

### RNA, DNA, and protein quantification

Cultured and thawed synovial MSCs and HUVECs (3 × 10^5^ cells) were suspended in 250 µL of 95% FBS and 5% DMSO (Fujifilm Wako Pure Chemical Corporation, Tokyo, Japan). Total RNA, DNA, and protein were extracted from the cells using an AllPrep DNA/RNA/Protein Mini Kit according to the manufacturer’s instructions (Qiagen N.V., Venlo, The Netherlands). RNA and DNA concentrations were measured using a NanoDrop 1000 instrument (Thermo Scientific). Protein concentrations were determined using a Pierce BCA protein assay kit (Thermo Fisher Scientific).

### Colony forming unit (CFU) assay

For colony formation assays, cells were plated at 100 cells per 9.6 cm^2^. The wells were stained with 1% crystal violet (Fujifilm Wako Pure Chemical Industries, Osaka, Japan) after 12 and 10 days for MSCs and HUVECs, respectively. To compare the number of colonies, 18 replicates and three independent experiments were performed. One representative well is shown, and the number of colonies was counted manually. The colonies were imaged using a microscope (BZ-X700, Keyence Co., Ltd., Osaka, Japan) for further quantification. Image J software (National Institutes of Health, MD, USA) was used to quantify the crystal violet-positive area. The violet color was defined by selecting one violet area and then selecting the Image/Adjust/Color threshold tool and the Sample button. The image was then converted into an 8-bit binary image, and the crystal violet-positive area was calculated.

### RNA-seq

Total RNA was extracted from synovial MSCs and HUVECs using an RNeasy Mini Kit (Qiagen N.V.). RNA sequencing was performed as described previously [25]. Briefly, the RNA concentration and quality were determined using a Quantus Fluorometer (Promega Co., WI, USA) and an Agilent 2100 Bioanalyzer, respectively. All samples had an RNA integrity number > 7. Sequencing libraries were prepared using the Agilent SureSelect Strand-Specific RNA Library Prep (for Illumina). Briefly, poly-A RNA was purified from 300 ng of total RNA per sample using oligo-dT magnetic beads. The libraries were amplified by PCR for 13 cycles and were then purified using AMPure XP beads. The libraries were sequenced on an Illumina HiSeq1500 system using single-end 50 bp reads.

To analyze the altered genes, Venn diagram-based analysis and GO (gene ontology) term enrichment analyses were performed. Venn diagrams were generated for genes that were increased by more than twofold after DMSO supplementation using Venny software. The representative GO terms from the top 15 or 20 gene clusters were revealed using the Database for Annotation, Visualization and Integrated Discovery (DAVID) [26].

Among the 30,134 genes analyzed by RNA-seq, antioxidant-related genes determined using hierarchical clustering analysis and the genes in the pathway GO1903561 (extracellular vesicles) are shown. A heatmap was generated for genes showing more than two copies.

### Cell membrane fluidity measurements

We used dimethyl-6-dodecanoyl- 2-naphthylamine (Laurdan, AdipoGen Life Science, CA, USA) as a probe for the quantitative evaluation of cell membrane fluidity [18, 27–29]. The laurdan powder was dissolved in DMSO (9 mM final), and the solution was diluted in RPMI 1640 medium (33 µM final concentration). Cells in the medium were incubated for 30 min at ambient temperature. Fluorescence images of cells were captured by a confocal microscope (TCS-SP8, Leica Microsystems, Wetzlar, Germany) equipped with a × 10 objective lens (*NA* = 0.75) at ambient temperature. For laurdan extraction, a 405-nm laser diode was utilized as a light source. To evaluate membrane fluidity as a generalized polarization (GP) value, the fluorescence intensities at 406–460 nm (I_406–460_) and 470–530 nm (I_470–530_) were obtained using a spectrum analyzer in the confocal unit. The GP values were defined as follows:

1 $$GP = \frac{{I_{406 - 460} - G \times I_{470 - 530} }}{{I_{406 - 460} + G \times I_{470 - 530} }}$$

and G values were calibration factors, defined as:

2 $$G = \frac{{GP_{meas} + 1}}{{GP_{meas} - 1}} \div \frac{{GP_{ref} + 1}}{{GP_{ref} - 1}}$$

As a reference GP value for laurdan, *GP*_*ref*_ = 0.207 was used [18, 29]. The measured GP of laurdan in DMSO solution (33 µM in final) was used for *GP*_*meas*_ by using *G* = 1 in Eq. 1*.* The histograms for GP frequency were calculated from images of at least 30 cells per donor.

### Measurement of glutathione (GSH) and superoxide dismutase (SOD)

GSH was determined using a total glutathione quantification kit (Dojindo Laboratories, Kumamoto, Japan) according to the manufacturer’s instructions. Briefly, the cells were cultured and harvested. Then, 1.0 × 10^6^ cells were lysed by adding 10 mM HCl, frozen and thawed twice, added 5% sheep serum albumin, and centrifuged. The supernatant was used for the GSH assay following the manufacturer’s protocol. The absorbance of the solution at 405 nm was measured using a plate reader (Infinite M200; Tecan, Männedorf, Switzerland).

Time course alteration of GSH was determined using cultured cells in a 96-well plate. The cells were fixed and stained at each time point using ThiolTracker™ (Thermo Fisher Scientific) and their fluorescence was measured using a plate reader. The relative fluorescence unit (RFU) values of GSH per well were standardized by counting the RFUs of propidium iodide (Miltenyi Biotech, Bergish Gladbach, Germany), and the standardized RFUs were calculated as RFUs of GSH relative to time 0. The cells were also stained with ThiolTracker and observed under a microscope (BZ-X700).

SOD activity was examined using the SOD assay kit‐WST (DOJINDO LABORATORIES), according to the manufacturer’s instructions. Briefly, cells were cultured, and 1.0 × 10^6^ cells were harvested. The cells were homogenized to break the cell membrane, PBS was added, and the samples were centrifuged. The supernatant was used for the SOD assay following the manufacturer’s protocol. The absorbance of the solution at 450 nm was measured using a plate reader.

### Observation and quantification of ROS

At 0 and 3 h after supplementation with 5% DMSO, the cells were fixed with 4% paraformaldehyde and stained using CellROX® Green Reagent (Thermo Fisher Scientific). The cells were counterstained with 4,6-diamidino-2-phenylindole (DAPI; Fujifilm Wako) and analyzed under a microscope. Time course alteration of GSH was determined for cultured cells in a 96-well plate using a plate reader. The cells were fixed and measured at each time point using CellROX® Green Reagent. The RFU values of ROS per well were standardized by counting the RFUs of DAPI, and the standardized RFUs were calculated as RFUs of ROS relative to time 0.

### Improving freeze–thaw tolerance through pre-treatment

Before cell freezing, a stearoyl-coA desaturase (*SCD*) 1 inhibitor (CAY10566, Cayman Chemical, MI, USA) was added to the culture medium of MSCs (2.5 µM) and HUVECs (25 µM) for 3 h. After cell detachment, the cells were counted and frozen at 3.0 × 10^5^ cells/250 µL in 90% FBS, 5% DMSO, and 5 mM GSH (Teva Takeda Pharma Ltd., Aichi, Japan). Freezing and thawing were performed using the same technique described above.

### Statistical analysis

Statistical analysis was performed using Prism 9 (GraphPad Inc., La Jolla, CA, USA) and R software (The R Foundation for Statistical Computing, Vienna, Austria). Each statistical test used is described in the respective figure legend. Statistical significance was set at* p* < 0.05.

## Results

### Freeze–thaw tolerance of MSCs and HUVECs

**Fig. 1:**
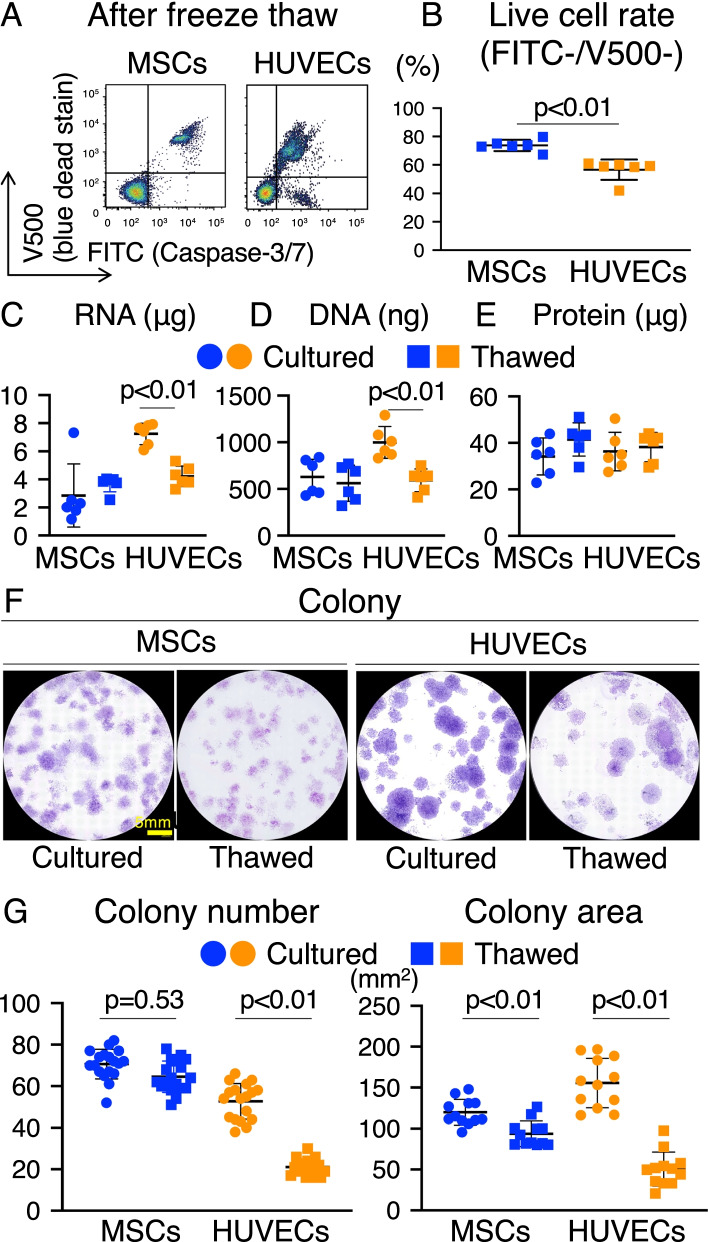
Freeze–thaw tolerance of MSCs and HUVECs. **a** Flow cytometric analysis of synovial MSCs and HUVECs after freeze-thawing. FITC-caspase-3/7 and V500-SYTOX™ blue dead cell staining is shown. **b** Live cell rate of synovial MSCs and HUVECs. Cells negative for FITC and V500 were considered live cells. Mean values and SD are shown (*n* = 6). The *p* value was calculated using the Mann–Whitney test. **c** RNA content in synovial MSCs and HUVECs (3.0 × 10^5^) before and after freeze-thawing. The mean values and SD are shown (*n* = 6). The *p* value was calculated using the Mann–Whitney test. **d** DNA content in synovial MSCs and HUVECs (3.0 × 10^5^) before and after freeze-thawing. The mean values and SD are shown (*n* = 6). The *p* value was calculated using the Mann–Whitney test. **e** Protein concentrations in synovial MSCs and HUVECs (3.0 × 10^5^) before and after freeze-thawing. The mean values and SD are shown (*n* = 6). The *p* value was calculated using the Mann–Whitney test. **f** Colony forming units (CFUs) of synovial MSCs and HUVECs before and after freeze-thawing. Representative dishes stained with crystal violet are shown. **g** CFUs after 12 days in culture before and after freeze-thawing. The mean values and SD are shown (*n* = 18). **h** Colony area after 12 days in culture before and after freeze-thawing. Mean values and SD are shown (*n* = 12). Cultured: fresh cells as seen before freeze-thawing. Thawed: cells immediately observed after freeze-thawing. The *p* value was calculated using the Kruskal–Wallis test with the Steel–Dwass multiple comparisons test

The live cell rate of synovial MSCs and HUVECs was examined after freeze-thawing (Fig. 1A). FITC^−^V500^−^ cells constituted approximately 75% of the MSCs and 60% of the HUVECs (Fig. 1B). Total DNA, RNA, and cellular protein yields from synovial MSCs and HUVECs were measured using cultured and thawed cells (Fig. 1C–E). In HUVECs, RNA and DNA content significantly decreased after freeze-thawing (Fig. 1C and D). In contrast, protein content in MSCs and HUVECs did not significantly decrease after freeze-thawing (Fig. 1E). Analysis of CFUs seeded immediately after freeze-thawing showed that the colony formation capacity of MSCs was maintained after freeze-thawing, whereas that of HUVECs was significantly reduced (Fig. 1F and G). The colony area was decreased in both cells.

Transcriptome analysis was performed to elucidate the alterations in gene expression profiles caused by freeze-thawing. The top 20 GO terms among 342 genes of MSCs and 542 genes of HUVECs were determined after freeze-thawing (Additional file 1: Fig. S1A). We found that 97 genes were commonly altered in the two cell types (Additional file 1: Fig. S1B). These 97 genes indicated that the alterations common to MSCs and HUVECs after freeze-thawing were mostly related to the extracellular matrix (Additional file 1: Fig. S1C).

### Alterations in gene expression profiles by DMSO supplementation

**Fig. 2:**
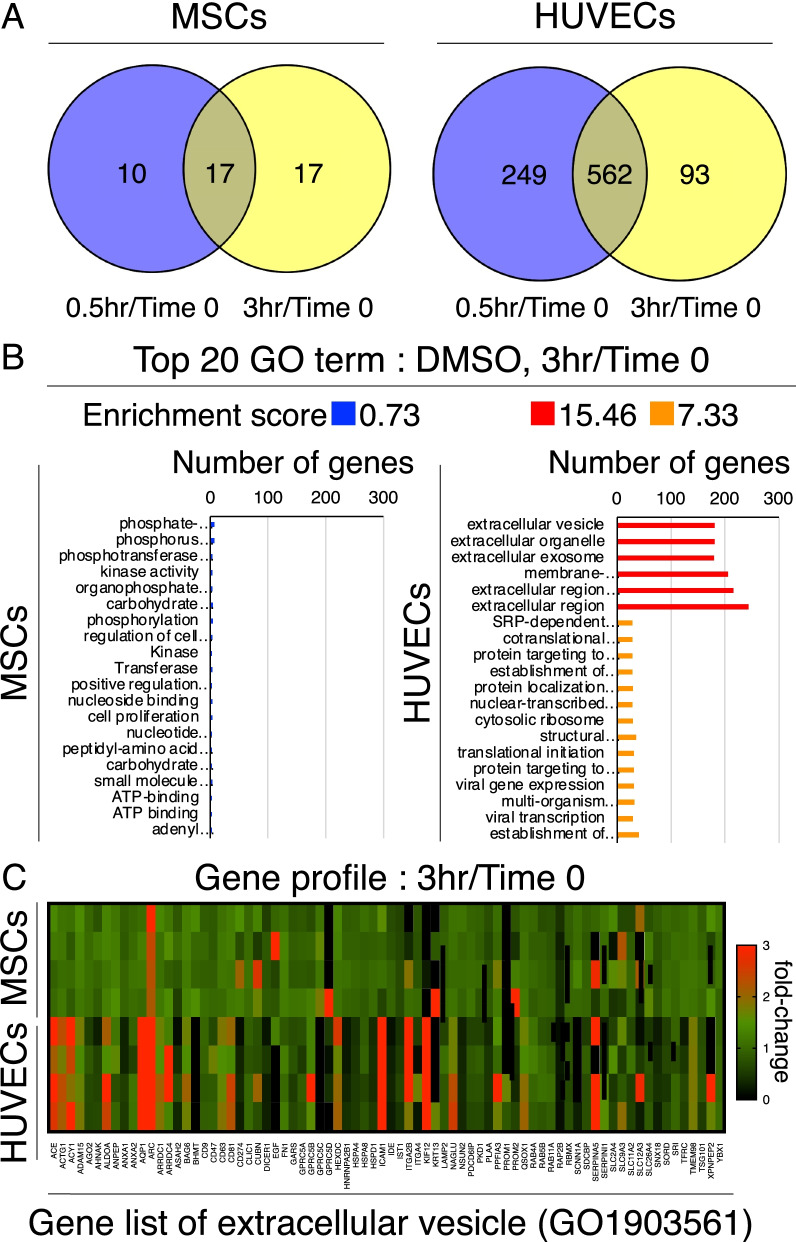
Alternations in gene expression profiles by DMSO supplementation. **a** Venn diagram of genes with twofold upregulation after DMSO supplementation. Time 0; before supplementation. **b** GO analyses for differentially expressed genes between before and after DMSO supplementation. The top 20 GO terms with high enrichment scores and low *p* values between the cells before and after DMSO supplementation are shown. **c** Heatmap for the GO1903561 (extracellular vesicle) gene profile of relative fold changes from time 0 to 3 h after DMSO supplementation

To evaluate the effect of DMSO use in the cryopreservation solution, alterations in gene expression were evaluated before and after DMSO supplementation. Venn diagram-based analysis of the transcriptome data revealed a higher number of genes upregulated in HUVECs between 0.5 and 3 h after supplementation with 5% DMSO (562 genes) compared to the overlap with MSCs (17 genes) (Fig. 2A). The expression of AQP1 and AQP5, also called water channels, was increased rapidly in HUVECs after adding 5% DMSO (Additional file 1: Fig. S2). The top 20 GO terms indicated enrichment of the extracellular vesicles and extracellular regions (Fig. 2B). A heatmap from the gene list of extracellular vesicles with the highest enrichment scores showed the fold change of gene expression before and 3 h after DMSO addition. Overall, significant alterations were observed in HUVECs compared to those in MSCs (Fig. 2C).

### Cell membrane fluidity before and after DMSO supplementation

**Fig. 3:**
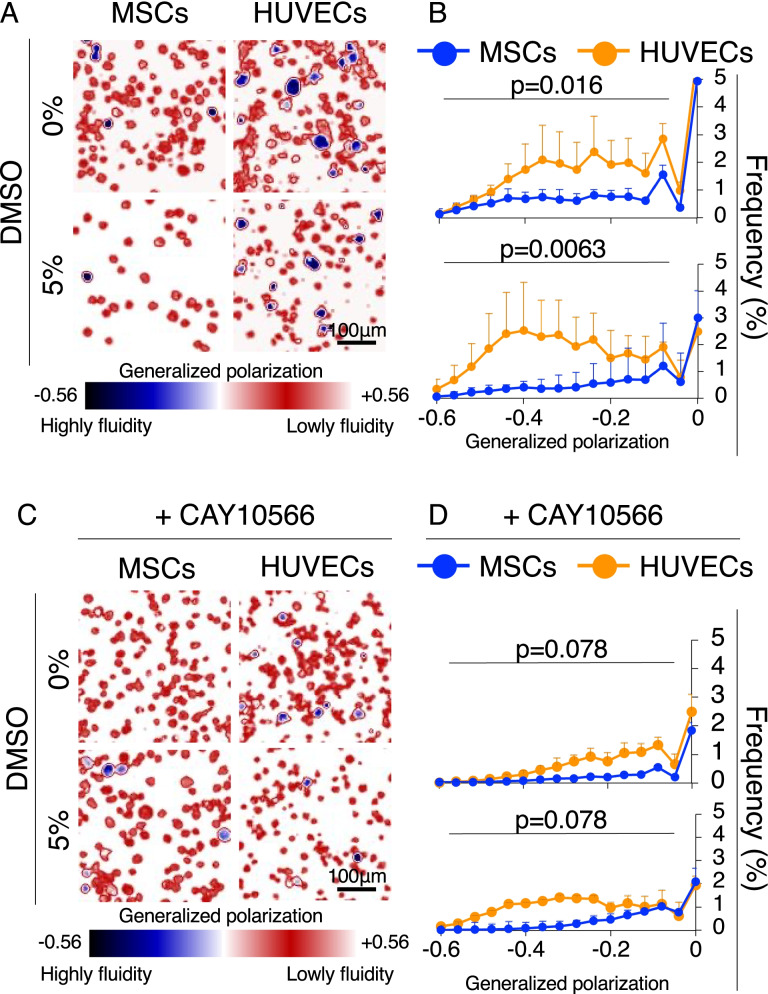
Cell membrane fluidity before and after DMSO supplementation. **a** Representative generalized polarization (GP) images of MSCs and HUVECs in the absence/presence of DMSO. **b** Histogram for GP frequency with mean values and SD (*n* = 3). The *p* value was calculated using the Kolmogorov–Smirnov test. **c** Representative GP images of MSCs and HUVECs in the absence/presence of DMSO and CAY10566 (stearoyl-coA desaturase 1 inhibitors). **d** Histogram for GP frequency showing the mean values and SD (*n* = 3). The *p* value was calculated using the Kolmogorov–Smirnov test

Based on the high expression of genes related to extracellular vesicle production, DMSO is considered to interact with the cell membrane as the first interface to tune its physical properties. To elucidate the physical action of DMSO, laurdan microscopy was applied to MSCs and HUVECs to measure membrane fluidity. First, HUVECs were found to have higher fluidity than MSCs (blue cells in Fig. 3A). Although the frequency and position of the first peak in the GP histogram (GP > 0.3) were comparable between MSCs and HUVECs (Additional file 1: Fig. S3), the frequency at the second peak of HUVECs in the high fluidity region (− 6 < GP < 0) was two times higher than that of MSCs (Fig. 3B). Further, the frequency of HUVECs in the high fluidity region was easily enhanced by adding 5% DMSO, whereas that of MSCs remained intact. Interestingly, high gene expression of stearoyl-CoA desaturase (*SCD1*), an enzyme that produces desaturated fatty acids, was observed in HUVECs compared to MSCs. Addition of a specific *SCD1* inhibitor (CAY10566) suppressed the appearance of cell membranes with high fluidity, even in the presence of DMSO (Fig. 3C). Statistical analysis of histograms clearly indicated that the inhibitor closed the gap in membrane fluidity signature between HUVECs and MSCs and increased tolerance to DMSO (Fig. 3D).

### Antioxidant capacity of MSCs and HUVECs

**Fig. 4:**
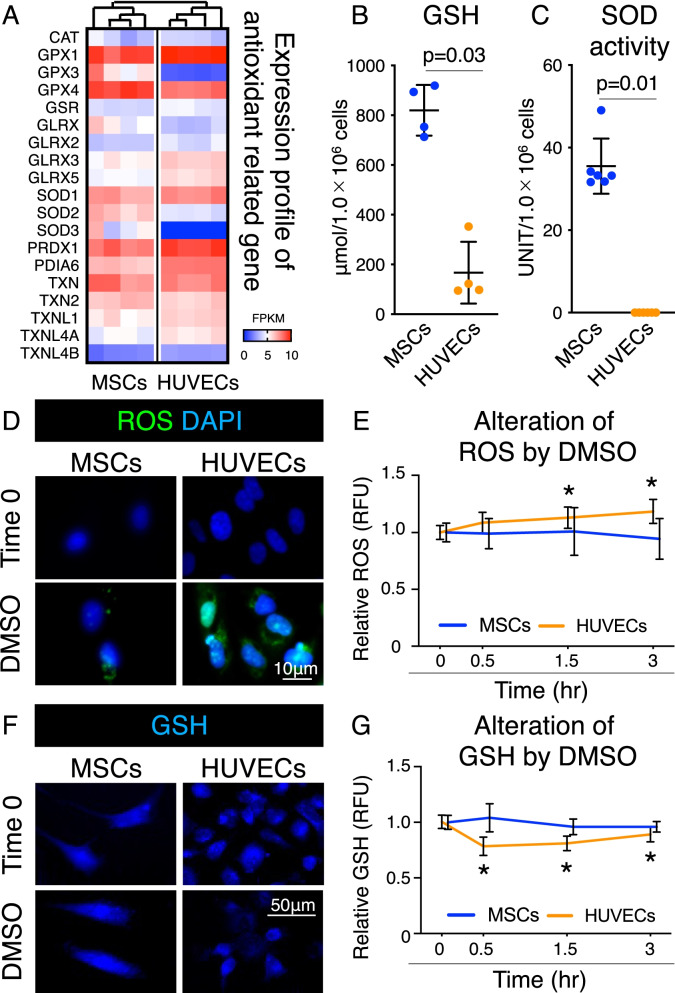
Antioxidant capacity of MSCs and HUVECs after DMSO supplementation. **a** Heatmap for expression profile of antioxidant-related genes. Cluster analyses are based on the expression profiles. FPKM: Fragments Per Kilobase of exon per Million mapped reads. **b** Amount of GSH in MSC and HUVEC lysates. Mean values and SD are shown (*n* = 4). The *p* value was calculated using the Mann–Whitney test. **c** Superoxide dismutase (SOD) activity in MSC and HUVEC lysates. Mean values and SD are shown (*n* = 6). The *p* value was calculated using the Mann–Whitney test. **d** Representative images of ROS (green) and cell nuclei stained with DAPI (blue). Data at time 0 and 3 h after supplementation with 5% DMSO are shown. **e** Alteration in ROS levels by 5% DMSO supplementation. Data are shown as relative fluorescence unit (RFU) values. *p < 0.05. The *p* value was calculated by two-way ANOVA with Dunnett’s multiple comparisons test. **f** Representative images of glutathione (GSH) (blue). Data at time 0 and 0.5 h after supplementation with 5% DMSO are shown. **g** Alterations in GSH levels by 5% DMSO supplementation. Data are shown as RFU values. *p < 0.05. The *p* value was calculated by two-way ANOVA with Dunnett’s multiple comparisons test

The expression profile of antioxidant-related genes showed that MSCs and HUVECs were characterized differently in the cluster analysis (Fig. 4A). Biochemical analysis revealed that GSH levels and SOD activities of MSCs were significantly higher than those of HUVECs (Fig. 4B), whereas the SOD activity of HUVECs was below the detection limit (Fig. 4C).

The effect of DMSO use in the cryopreservation solution on ROS production was also assessed. ROS production was observed in MSCs and HUVECs before adding 5% DMSO at time 0 and after 3 h. Compared to MSCs, HUVECs treated with 5% DMSO showed more ROS production after 3 h (Fig. 4D). Furthermore, variations in ROS production were not statistically significant in MSCs, whereas ROS production was significantly increased in HUVECs after 1.5 and 3 h (Fig. 4E). GSH levels in MSCs and HUVECs were observed after supplementation with 5% DMSO both at 0 and 0.5 h; however, cells with low fluorescence intensity were present only in HUVECs (Fig. 4F). GSH levels changed over time with the addition of 5% DMSO, but not significantly, in MSCs. On the contrary, GSH levels significantly decreased after 0.5 h and remained low after 1.5 h and 3 h in HUVECs (Fig. 4G).

### Improving freeze–thaw tolerance through pre-treatment

**Fig. 5:**
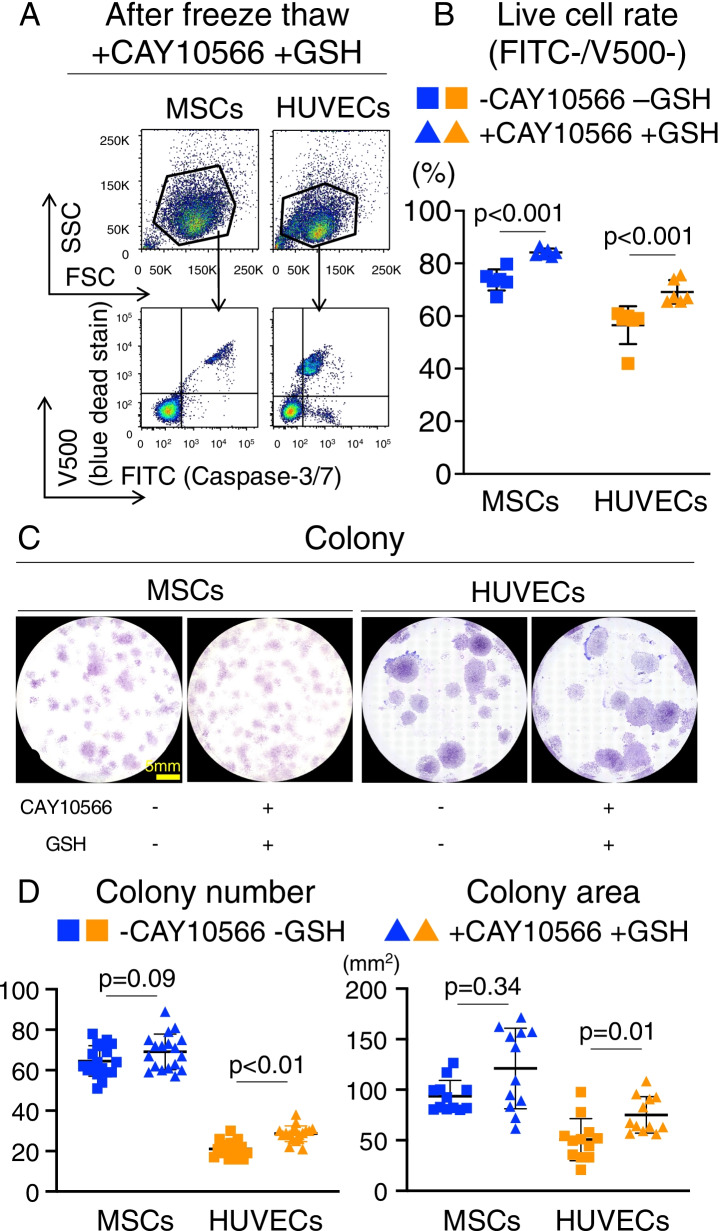
Improving freeze–thaw tolerance through pre-treatment. **a** Flow cytometric analysis of synovial MSCs and HUVECs after freeze-thawing by forward scatter (FSC) and side scatter (SSC), stained with FITC-caspase-3/7 and V500-SYTOX™ blue dead cell stain. **b** Live cell rate of synovial MSCs and HUVECs. Cells negative for FITC and V500 were considered live cells. Mean values and SD are shown (*n* = 6). The *p* value was calculated using the Mann–Whitney test. **c** Colony forming units (CFUs) of synovial MSCs and HUVECs after freeze-thawing. Representative dishes stained with crystal violet. **d** CFUs after 12 days in culture before and after freeze-thawing. Mean values and SD are shown (*n* = 18). **e** Colony area after 12 days in culture before and after freeze-thawing. Mean values and SD are shown (*n* = 12). Thawed: cells immediately after freeze-thawing. The *p* value was calculated using the Kruskal–Wallis test with Steel–Dwass multiple comparisons test

After controlling cell membrane fluidity using CAY10566 and reducing the damage from ROS using GSH, MSCs and HUVECs were evaluated for live cell rate after freeze-thawing. The combination of CAY10566 and antioxidant GSH treatment significantly improved the ratio of FITC^−^V500^−^ cells immediately after freeze-thawing from 74 ± 4% to 84 ± 1% and 57 ± 7% to 69 ± 4% in MSCs and HUVECs, respectively (Fig. 5A and B). Analysis of CFUs seeded immediately after freeze–thaw treatment showed that the colony formation capacity of HUVECs was increased after treatment with CAY10566 and GSH (Fig. 5C and D). The colony area was also significantly increased in HUVECs (Fig. 5C and D). These results clearly indicate that controlling the physical and biological properties of cells can improve freeze–thaw tolerance.

## Discussion

We found that differences in resistance to DMSO between MSCs and HUVECs is a biological and physical factor controlling freezing tolerance. DMSO protects cells by making the preservation solution highly osmotic, thereby removing water from cells and preventing the formation of ice crystals [10–12]. However, some cell types are severely impaired by DMSO function. HUVECs showed lower freeze–thaw tolerance than MSCs, and the variation in gene expression was significantly affected by DMSO addition. Analysis of cell membrane fluidity showed a larger number of cells with high fluidity among HUVECs than among MSCs, and fluidity was easily enhanced by the addition of DMSO. HUVECs were also less tolerant to ROS generation, which is known to be one of the common toxicity mechanisms of DMSO. Considering the low activities of GSH and SOD in HUVECs, which reduce ROS, DMSO can easily cause cytotoxicity. The freeze–thaw tolerance of HUVECs was improved by (1) controlling their cell membrane fluidity, thus reducing the effect of DMSO, and (2) suppressing the internal ROS-driven cytotoxicity using GSH.

Unlike MSCs, HUVECs showed significantly decreased live cell rate, colony forming ability, and colony area after freeze-thawing. Thus, HUVECs were more negatively affected by freeze-thawing than MSCs. Cell viability is generally reduced by freeze-thawing in any cell type. Water influx into cells is one of the main factors for cryoinjury. Cryoprotectants accelerate water loss; however, excessive water loss causes shrinkage of cells below a lethal minimum volume [30]. The water permeability of cells is dependent on the cell type [31, 32]. The water permeability of MSCs and HUVECs is affected by their original in vivo environment. Conventionally, synovial MSCs exist in the knee joint with abundant joint fluid, which is a highly osmotic environment in the body due to the presence of an extracellular matrix (*e.g.*, hyaluronic acid). Therefore, the cell membrane of MSCs may originally have high osmotic tolerance and low water permeability. The osmolality of blood, where HUVECs are found, is 280 mOsm/kg H_2_O, whereas that of the joint fluid harboring synovial MSCs is 400 mOsm/kg H_2_O [33, 34]. In vivo, synovial MSCs are always exposed to high osmotic pressure from joint fluid and may originally be tolerant to the hyperosmotic environment formed by DMSO, which has an osmolality of 700–1000 mOsm/kg H_2_O. Therefore, MSCs may inhibit excessive water influx and minimize injury after freeze-thawing.

Although DMSO is the most common cryoprotectant, its toxicity has been indicated in various studies [13, 14]. In the present study, the alteration of gene expression at 30 min and 3 h after addition of 5% DMSO was analyzed in MSCs and HUVECs. The expression of a large number of genes was altered in HUVECs, whereas fewer gene changes were observed in MSCs. Furthermore, the expression of the water channels, AQP1 and AQP5, was increased in HUVECs, suggesting that a large amount of water loss and/or influx occurred in HUVECs [35]. GO term analysis showed that the fluctuating genes were mainly related to the extracellular region, indicating that a large amount of intracellular water loss and excessive cell shrinkage induced significant extracellular-related gene changes upon DMSO addition.

GO term analysis indicated that extracellular vesicle-related genes constituted the most altered gene set in HUVECs upon DMSO addition. Extracellular vesicles are bilayered lipid membrane particles that carry specific proteins or RNAs and are released upon various external stimuli. The release of extracellular vesicles by murine BV-2 microglial cells and astrocytes is increased by ethanol treatment, and the content of the vesicles is enriched in inflammatory-related proteins and miRNAs related to immune response activation [36, 37]. Therefore, extracellular vesicles are also expected to play a role as biomarkers of disease states [38]. In this study, small vesicle-like structures were observed around HUVECs after DMSO addition. Similar to the response of rat hepatocytes to ethanol treatment [39], the HUVEC membrane fluidity and ROS generation were altered upon DMSO addition. Hence, the increased expression of extracellular vesicle-related genes may be a secondary response caused by excessive water loss by the cell and shrinkage.

DMSO interacts with the cell membrane as the first interface. Indeed, DMSO is known to have a significant effect on the cell membrane, for example, by creating pores in the phospholipid bilayers and altering the water permeability of the cell membrane [40–43]. However, the correlation between cell membrane fluidity and DMSO reactivity in MSCs and HUVECs has not been fully elucidated. In the present study, MSCs showed lower cell membrane fluidity than HUVECs. The membrane fluidity of HUVECs was enhanced by adding 5% DMSO, whereas the membrane fluidity of MSCs was not significantly affected. An increase in membrane fluidity has been considered to cause pore formation in the membrane, facilitating the influx of extracellular solutes [44, 45]. This excessive extracellular solute entry may have caused larger gene expression changes in HUVECs responding to DMSO, as their fluidity is different from that of MSCs. Cell membrane fluidity is significantly influenced by the expression level of stearoyl-coA desaturase (*SCD1*) [18, 46, 47] (Additional file 1: Fig. S4). Regulation of membrane fluidity in HUVECs via inhibition of *SCD1* by CAY10566 resulted in the membrane fluidity of HUVECs being comparable to that of MSCs. After CAY10566 treatment, the membrane fluidity of HUVECs in response to DMSO was significantly suppressed. These results clearly suggest that HUVEC membrane fluidity is regulated by *SCD1*, and that inhibition of *SCD1* leads to DMSO tolerance.

Differences in cell membrane fluidity between different cell types are closely related to their characteristics. For example, a comparison between undifferentiated and differentiated cells, from induced pluripotent stem cells to endodermal cells, revealed that differentiated cells have lower membrane fluidity [18]. In this study, we focused on the relationship between the original characteristics of each cell type and freeze–thaw tolerance. Differentiation changes the properties of cells, possibly affecting their freeze–thaw tolerance. Therefore, evaluating the original properties and freeze–thaw tolerance of each cell type will provide important information for the design of a manufacturing protocol for therapeutic cells.

ROS generation, a toxic effect of DMSO, is highly damaging to cells [48, 49]. In the current study, the amount of ROS generated by MSCs and HUVECs differed due to differences in antioxidant capacity; MSCs possessed high antioxidant capacity, as reported in previous studies [50–52]. HUVECs had low antioxidant capacity and were possibly damaged by ROS. Decreasing the DMSO concentration could reduce the amount of ROS generated; however, it would not be sufficient to maintain the osmotic pressure required for freezing, resulting in greater damage from intracellular ice formation. Several researchers have attempted to improve the viability of cells by adding antioxidants (*e.g.*, GSH and catalase) to the cryopreservation solution for suppressing ROS generation [53–55]. Further, strict temperature control using programmed freezers also attenuates intracellular ice formation [5]. To clarify resistance to DMSO, it is important to preliminarily evaluate the antioxidant properties of target cells before cryopreservation, considering both the physical and biological aspects.

Pre-treatment with CAY10566 (*SCD1* inhibitor) and addition of GSH (antioxidant) to the cryopreservation solution was found to significantly improve freeze–thaw tolerance. These treatments improved the survival rates of MSCs and HUVECs after freeze-thawing. In particular, colony forming ability and colony area of HUVECs were significantly improved, whereas MSCs showed a tendency to improve, but not significantly. GSH, one of the most abundant low-molecular-weight antioxidants, has the ability to remove ROS [56], which may also have improved the viability of HUVECs in the current study. CAY10566 is an inhibitor of *SCD1*, which catalyzes the biosynthesis of saturated fatty acids to monounsaturated fatty acids. The fluidity of cell membranes possibly reflects the amount of saturated to monounsaturated fatty acids; thus, the expression level of *SCD1* contributes to cell membrane fluidity [57]. In the current study, altering the components of the phospholipid bilayer of HUVECs resulted in a fluidity comparable to that of MSCs. Our results suggest that these changes suppressed the action of DMSO and improved cell survival.

This study had three limitations. First, the improvement in cell viability was limited and was not recovered to the cell viability level before freezing. It is thus possible that other factors may have influenced freeze–thaw tolerance. Cell surface biophysical properties are a candidate for influencing resistance to the ice crystals produced during freezing. For example, a factor directly related to ice crystal formation is proposed as the physical distance of the gap junction between cell membranes [58]. To understand the freeze–thaw tolerance of cells, it is necessary to analyze the molecular mechanisms as well as the biophysics of cells. Second, no cytoskeleton analysis was performed in this study. The cytoskeleton is known to be disrupted after freeze-thawing [59]. Alterations in the cytoskeleton such as blebbing have also been observed due to freeze-thawing and osmotic responses [60, 61]. Using a Rock inhibitor (Y27632), acting disruption has been shown to decrease, and this treatment improves survival rate after freeze-thawing [62]. Therefore, additional combination of a Rock inhibitor targeting the cytoskeletal effects in HUVECs may effectively improve the cell survival rate. Third, in addition to CAY10566, several other reagents can control cell membrane fluidity. For example, epigallocatechin gallate (EGCG) is an antioxidant [63, 64] that can decrease cell membrane fluidity [65, 66]. In human osteosarcoma cells, EGCG was found to suppress the growth inhibition that occurs during repeated freeze–thaw cycles [67]. Therefore, freeze–thaw tolerance may be improved by evaluating various reagents that control cell membrane fluidity.

## Conclusion

**Fig. 6:**
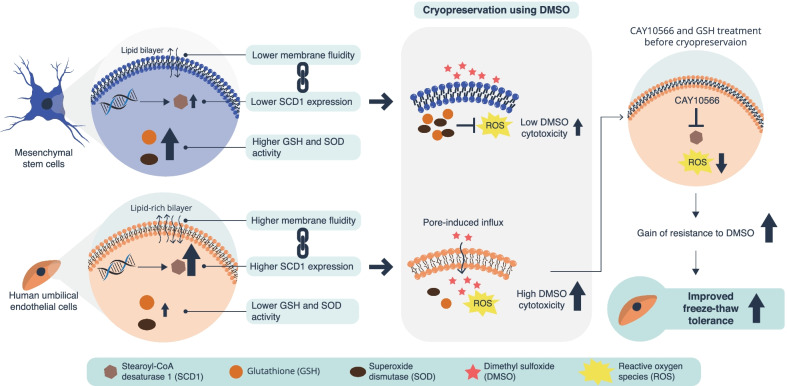
Graphical abstract. Decreasing the toxicity of dimethyl sulfoxide (DMSO) as a cryoprotectant can be achieved by controlling cell membrane fluidity and reducing reactive oxygen species (ROS)-induced damage. This can improve the freeze–thaw tolerance of HUVECs

We found that MSCs and HUVECs differed in their resistance to DMSO when used as a cryoprotectant, and that their DMSO tolerance depended on cell membrane fluidity and ROS resistance (Fig. 6). The findings suggest that DMSO acts on the lipid-rich cell membrane of HUVECs, increasing the fluidity of the cell membrane. This higher fluidity leads to the formation of a pore-induced DMSO influx into the cytoplasm, which enhances ROS production and induces greater cytotoxicity in HUVECs, which have lower antioxidant capacity than MSCs. In conclusion, cell-specific differences in freeze–thaw tolerance originate from differences in cell membrane fluidity and antioxidant capacity. By demonstrating the efficacy of analyzing the freeze–thaw tolerance of cells from the viewpoint of both biology and membrane-physics, our findings provide a basis for establishing appropriate cryopreservation procedures and promoting cell transplantation therapies.

## Supplementary Information

**Additional file 1: Figure S1**. Alteration of mRNA expression profiles after freeze-thawing. (a) GO analyses for differentially expressed genes between cells before and after freeze-thawing. The top 15 GO terms with high enrichment scores and low *p* values between freeze-thawed and fresh cells are shown. (b) Venn diagram of genes with twofold upregulation after freeze-thawing in MSCs and HUVECs. (c) GO terms with high enrichment scores and low *p* values for the 97 co-expressed genes in MSCs and HUVECs. **Figure S2**. Differences in AQP expression after supplementation with 5% DMSO between MSCs and HUVECs. **Figure S3**. Cell membrane fluidity. (a) Low magnification histograms for frequency by GP are shown with mean ± SD (*n* = 3). Data are shown for conditions before and after DMSO supplementation. (b) Low magnification histograms for frequency by GP are shown with mean ± SD (*n* = 3). Data are shown in the absence/presence of DMSO and CAY10566 (stearoyl-coA desaturase 1 inhibitors). **Figure S4**. Differences in lipogenesis between MSCs and HUVECs.

## Data Availability

The data set supporting the results of RNA-sequence is available in the Open Science Framework repository: 10.17605/OSF.IO/VF2MR.

## Abbreviations

MSC: Mesenchymal stem cell
HUVEC: Human umbilical vein endothelial cell
DMSO: Dimethyl sulfoxide
SCD1: Stearoyl-coA desaturase
GSH: Glutathione
ROS: Reactive oxygen species
RFU: Relative fluorescence unit
CFU: Colony forming unit
GP: Generalized polarization
SOD: Superoxide dismutase
GO: Gene ontology
AQP: Aquaporin
EGCG: Epigallocatechin gallate
