# Supplementary figures and images for: Cell membrane fluidity and ROS resistance define DMSO tolerance of cryopreserved synovial MSCs and HUVECs
Source: Stem Cell Res Ther. 2022 May 3;13:177. doi: 10.1186/s13287-022-02850-y (PMC9066911; doi:10.1186/s13287-022-02850-y)

Figure S1

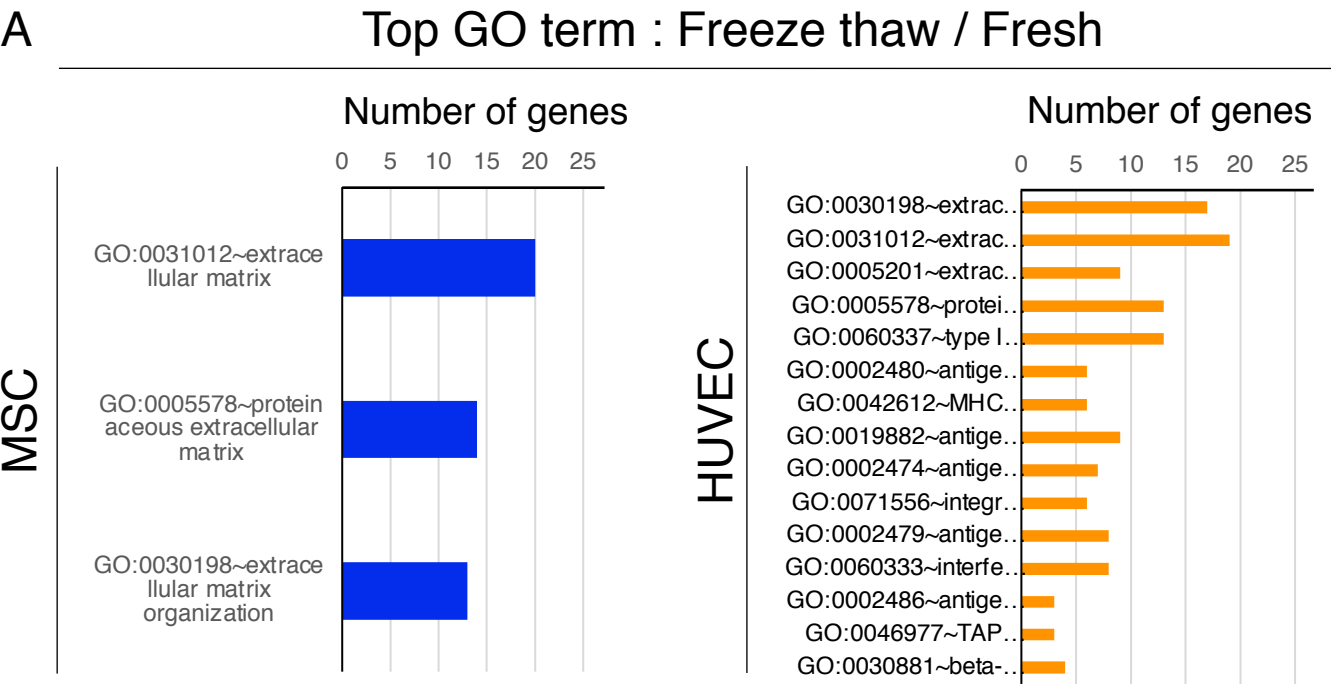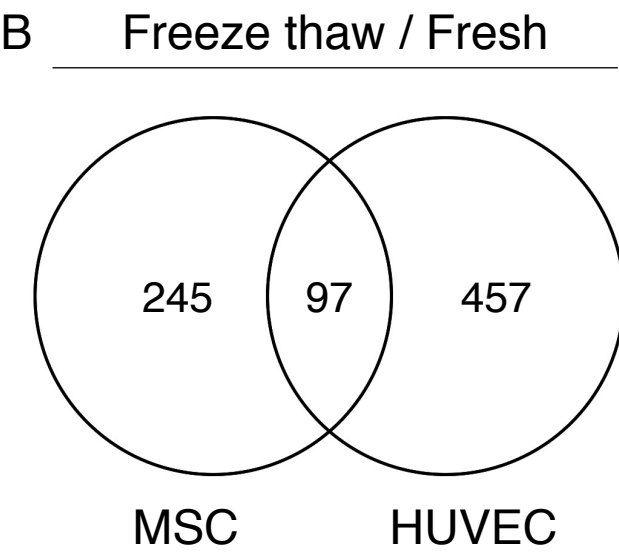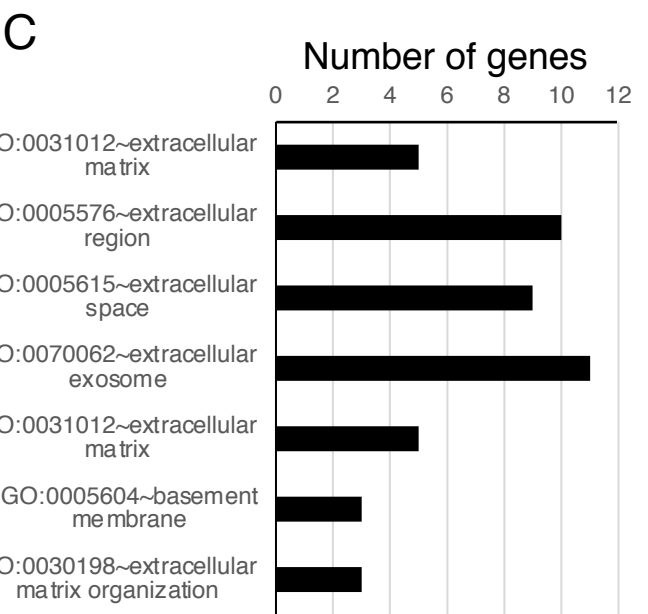

Figure S2

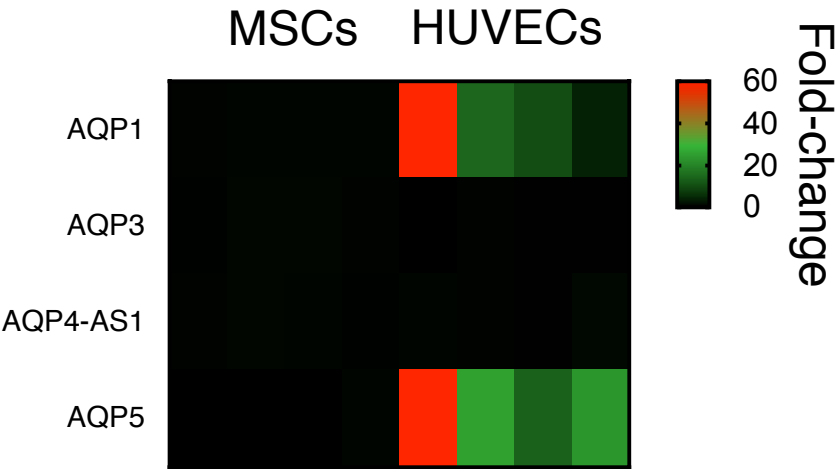

Figure S3

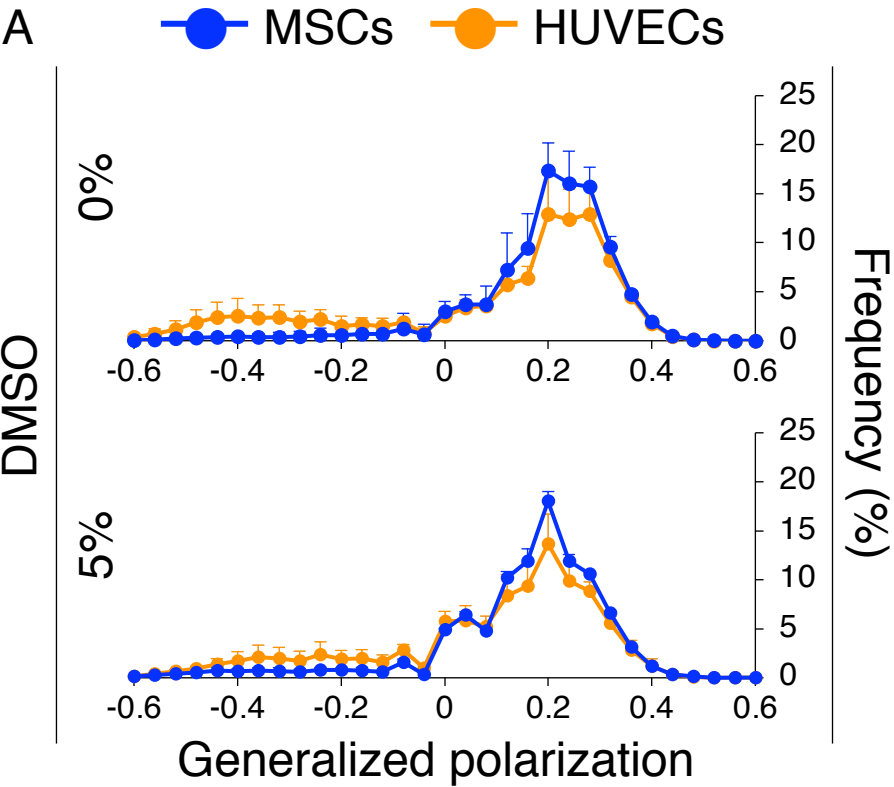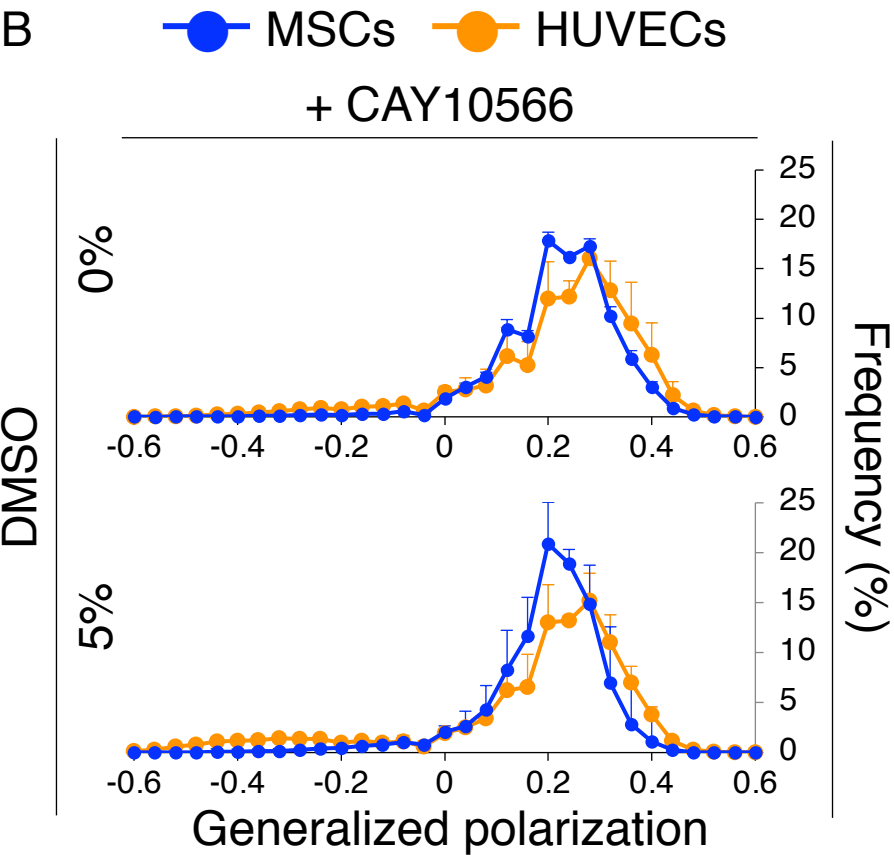

Figure S4

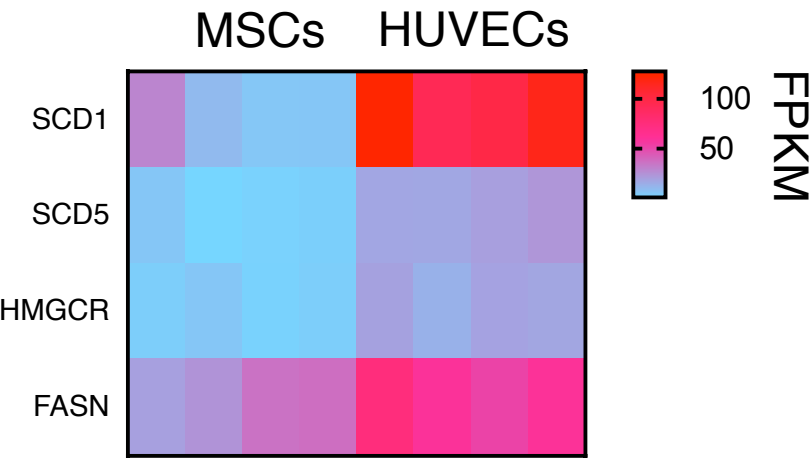

Supplement: Supplementary file 1 — Additional file 1: Figure S1. Alteration of mRNA expression profiles after freeze-thawing. (a) GO analyses for differentially expressed genes between cells before and after freeze-thawing. The top 15 GO terms with high enrichment scores and low p values between freeze-thawed and fresh cells are shown. (b) Venn diagram of genes with twofold upregulation after freeze-thawing in MSCs and HUVECs. (c) GO terms with high enrichment scores and low p values for the 97 co-expressed genes in MSCs and HUVECs. Figure S2. Differences in AQP expression after supplementation with 5% DMSO between MSCs and HUVECs. Figure S3. Cell membrane fluidity. (a) Low magnification histograms for frequency by GP are shown with mean ± SD (n = 3). Data are shown for conditions before and after DMSO supplementation. (b) Low magnification histograms for frequency by GP are shown with mean ± SD (n = 3). Data are shown in the absence/presence of DMSO and CAY10566 (stearoyl-coA desaturase 1 inhibitors). Figure S4. Differences in lipogenesis between MSCs and HUVECs. [file 13287_2022_2850_MOESM1_ESM.pdf]
